# Rethinking chronic care: how blended patient-centered care delivery and innovative financing models can contribute to achieving universal health coverage—a case study of an integrated approach in Kenya

**DOI:** 10.1093/oodh/oqaf002

**Published:** 2025-01-12

**Authors:** Judith van Andel, Gloria P Gómez-Pérez, Peter Otieno, Angela Siteyi, Julia Teerling, Tobias Rinke de Wit, Gershim Asiki

**Affiliations:** PharmAccess, Paasheuvelweg 25, 1100 DE Amsterdam, The Netherlands & Nairobi, Kenya; Amsterdam Institute of Global Health and Development, Paasheuvelweg 25, 1100 DE Amsterdam, The Netherlands; PharmAccess, Paasheuvelweg 25, 1100 DE Amsterdam, The Netherlands & Nairobi, Kenya; Amsterdam Institute of Global Health and Development, Paasheuvelweg 25, 1100 DE Amsterdam, The Netherlands; African Population Health Research Center, Manga Close, Kirawa road, Nairobi, Kenya; PharmAccess, Paasheuvelweg 25, 1100 DE Amsterdam, The Netherlands & Nairobi, Kenya; PharmAccess, Paasheuvelweg 25, 1100 DE Amsterdam, The Netherlands & Nairobi, Kenya; PharmAccess, Paasheuvelweg 25, 1100 DE Amsterdam, The Netherlands & Nairobi, Kenya; Amsterdam Institute of Global Health and Development, Paasheuvelweg 25, 1100 DE Amsterdam, The Netherlands; African Population Health Research Center, Manga Close, Kirawa road, Nairobi, Kenya

**Keywords:** non-communicable disease (NCD), hypertension, diabetes, digital health, low-middle income countries (LMIC), universal health coverage (UHC), home-monitoring, self-management, digital financing

## Abstract

Universal Health Coverage (UHC) aims to ensure all individuals have access to essential health services without financial hardship. Chronic diseases, like hypertension and diabetes, play a critical role in achieving UHC due to their lifelong management needs. This paper examines the implementation of a digital and mobile-based, patient-centered care model aimed at improving care for hypertensive and diabetic patients in Kenya. Between 2018 and 2019, 1626 patients from nine clinics in Nairobi, Kiambu, Nyeri and Vihiga counties were enrolled in an integrated non-communicable disease (NCD) care model including self-management devices for home monitoring, a digital health wallet (M-TIBA) for co-payment and facility-based peer support groups. Follow-up data was collected November–December 2020. Results indicated significantly improved patient outcomes, with 50% of hypertensive and 74% of diabetic patients achieving disease control, compared to 42% and 52% at baseline. Additionally, peer group participation increased adherence to self-monitoring and lifestyle modifications, contributing to better health outcomes. Despite these successes, challenges such as accessing medications and technical issues with digital tools were identified. Financial sustainability and scalability remain critical concerns, particularly in under-resourced settings. The case study highlights the potential of digital health solutions to enhance chronic care and support UHC by improving accessibility and reducing costs. A multifaceted approach, combining digital tools with face-to-face support and addressing structural barriers in healthcare systems, is essential for long-term success. The findings contribute to the broader discourse on integrated care models for NCDs in low-resource settings, underscoring the importance of sustainable financing and innovative care delivery mechanisms.

## INTRODUCTION

### NCDs: a chronic emergency

Low- and middle-income countries (LMICs) face a growing burden of non-communicable diseases (NCDs). NCDs account for a significant 74% of global deaths, impacting ~41 million people annually. Notably, 17 million of these deaths occur prematurely among individuals aged 30 to 69, with an overwhelming 86% of these early fatalities happening within LMICs. Projections also suggest that NCDs are on track to surpass communicable diseases, maternal and perinatal conditions and nutritional disorders as the leading global causes of death by 2030. In LMICs, cardiovascular diseases (CVDs), are major contributors to NCD-related mortality, constituting a significant 54% of disability-adjusted life-years. Within this complex landscape, diabetes and hypertension are pivotal cardiovascular risk factors, with hypertension being the leading metabolic risk factor to which 19% of global deaths are attributed, followed by raised blood glucose (BG) and overweight and obesity [1].

The above statistics do not paint the full picture—an estimated 52% of women and 66% of men with hypertension in Sub Sahara Africa (SSA) remain undiagnosed. Those diagnosed are often untreated or treated unsuccessfully with 13% of women with hypertension and 9% of men with hypertension reaching controlled blood pressure (BP) levels [2]. The already underfunded and understaffed healthcare systems are not able to cope, especially because chronic diseases require long-term medication and management. For patients that do seek and receive treatment, out-of-pocket costs are high and treatment can often lead to catastrophic health expenditure [3].

In Kenya, prevalence rates are high with varying diagnosis and treatment rates between rural and urban settings. The prevalence of hypertension in the adult population is around 25–29% [4–6] while diabetes affects around 2–5% [7–9]. The prevalence of hypertension seems similar in both urban and rural areas, while diabetes has higher prevalence in urban regions compared to rural areas [4, 9]. Awareness and treatment rates vary, but generally seem lower in rural areas and urban slum areas [6, 7, 10, 11], with awareness levels for hypertension as low as 12% reported in lower wealth quintiles and fewer than 20% of those receiving effective treatment [4]. In urban areas, particularly slums, challenges in treatment adherence are reported, with over 75% of patients failing to attend follow-up appointments [12]. Rural health systems are ill-equipped to handle the growing burden of these non-communicable diseases, necessitating an urgent reorientation of care infrastructure [13]. Overall, both settings struggle with high prevalence and low awareness and treatment rates but face unique barriers to effective treatment and control in terms of differing lifestyle patterns, availability of health facilities and trained personnel and costs of healthcare.

### NCDs in the context of universal health coverage

Universal Health Coverage (UHC) is a fundamental principle that aims to ensure that all individuals and communities have access to essential health services without suffering financial hardship. At its core, UHC embodies the idea that every person should receive the necessary healthcare they need to maintain good health, prevent illnesses and receive appropriate treatment when needed, regardless of their socio-economic status or geographical location. Achieving UHC goes beyond mere access to medical services; it encompasses a comprehensive approach to healthcare that includes promotion, prevention, treatment and rehabilitation (WHO fact sheet UHC, 2023) [14].

Chronic care plays a pivotal role in the realization of Universal Health Coverage. Chronic diseases, such as diabetes, cardiovascular diseases and respiratory conditions, have become significant contributors to the global burden of disease. Unlike acute illnesses, chronic conditions require continuous, often lifelong, management and care. As a result, the effective management of chronic diseases is essential not only for improving individual well-being but also for the sustainability of healthcare systems at large and mitigating the economic impact of preventable complications.

To reach UHC and improve the lives of patients affected by these diseases, and avoid a further overburdening of the healthcare system, we need to fundamentally rethink how healthcare delivery for chronic non-communicable diseases is organized and financed. In this paper we provide context on challenges for chronic care delivery and present our experience with a case study implementing an integrated NCD care-model with an innovative blended care and financing approach in Kenya.

## CURRENT CHALLENGES IN CHRONIC CARE DELIVERY

Access to chronic care in resource-restricted settings faces significant challenges, as highlighted by various research papers. These challenges encompass a range of factors affecting healthcare systems, patients and communities.

### Healthcare Infrastructure and Health workforce shortages

Limited healthcare infrastructure in low-resource settings, hinders access to chronic care [15–17]. Insufficient facilities, medical equipment and diagnostic tools result in delayed or inadequate treatment and weak health information systems, hinder patient tracking and care coordination, leading to fragmented care [18–20]. Workforce shortages further contribute to the challenge. A lack of trained healthcare professionals, including doctors and nurses, reduces the availability of care providers for chronic disease management.

### Medication accessibility

Several papers [19, 21] highlight issues related to medication access. High costs, limited availability and supply chain disruptions create barriers to obtaining essential medications for chronic conditions.

### Patient education

Low health literacy and limited awareness, [15, 18, 22], impede access to chronic care. Patients may not recognize symptoms, seek care late or struggle with self-management due to insufficient understanding of their conditions and risks.

### Socioeconomic, geographical, cultural and behavioral factors

The same references mention socioeconomic determinants to play a significant role in access disparities. Poverty, lack of transportation and competing priorities prevent individuals from accessing and adhering to chronic care. Patients in remote areas may face travel challenges and limited healthcare facilities. Cultural beliefs and behaviors can affect access: stigma and traditional healing practices may discourage patients from seeking Western healthcare for chronic conditions.

### Health system priorities, policy and funding

Health system priorities that favor acute care over chronic care, result in delayed diagnosis and insufficient resources for managing chronic conditions. Policy gaps and inadequate funding constrain the development and implementation of comprehensive chronic care programs.

These challenges underscore the need for a multifaceted approach that addresses healthcare system deficiencies, improves patient education, considers socioeconomic disparities and promotes policy changes to prioritize chronic care. Effective solutions should also consider the unique cultural contexts in which chronic care is provided.

## INTEGRATED CHRONIC CARE DELIVERY MODELS

A widely recognized framework for such a multifaceted approach is the Wagner Chronic Care Model designed to enhance the quality of chronic care delivery with an integrated approach. It consists of six key components, including health care organization, self-management support, delivery system design, decision support, clinical information systems and community resources. The model emphasizes health care organization to support a patient-centered approach, proactive care and care teams working together to manage chronic conditions effectively. Self-management support encourages patients to actively engage in managing their health, while delivery system redesign ensures that care is organized to meet the unique needs of individuals with chronic diseases. Decision support provides healthcare providers with the latest evidence-based guidelines and can guide timely referral, and clinical information systems aid in tracking patient data, facilitating better decision-making. Lastly, community resources are leveraged to support patients’ long-term management of chronic conditions [23]. The implementation of elements of the Chronic Care Model has shown promise in improving outcomes of chronic care in various settings. Two meta-analyses evaluating the available evidence for integrated approaches to chronic care in LMICs can contribute to better health outcomes for patients with hypertension and diabetes, though robustness of evidence is often low. Very few of the identified studies addressed more than two elements of the chronic care model in their integrated approach, due to challenges mentioned in the previous paragraph [24, 25].

## CASE STUDY: A DIGITAL AND MOBILE-PHONE-BASED, PATIENT-CENTERED CARE MODEL FOR HYPERTENSIVE AND DIABETIC PATIENTS IN KENYA

### Care model

An integrated NCD outpatient care-model based on a mobile platform was setup for which in total 1.626 patients with hypertension and/or diabetes were registered between September 2018 and September 2019 from selected clinics in Nairobi, Kiambu and Vihiga county in Kenya. The NCD care-model consisted of:

- Self-management devices distributed to patients to measure BP or BG levels at home. The distributed devices were of various models, depending on what was available through the clinic attended by the patients. All devices were approved by the Kenya Pharmacy and Poisons Board. The patients were trained to use their devices by a trained nurse at the clinic and to take their measurements at home and enter their readings on a mobile phone application (AfyaPap, developed by Baobab Circle©), to relay their measurements to their healthcare provider. In addition, health education messages were shared with patients through the AfyaPap application. The application was available on smartphone and in a USSD-menu format for feature phones (see supplementary Fig. 1 for an overview of the application features). If any issues arose with their measurement device or application patients could seek support through phone or at the clinic and/or support group they attended. No proactive quality checks of the devices were performed through the clinics or research team.
- A mobile health wallet (M-TIBA, by CarePay©) that gave access to a co-payment model for diabetes and hypertension consultations, medical tests and medicines at a selection of clinics and allowed patients to save for health care payments. Twenty percent of costs were covered with patients’ individual funds and 80% through program funds. Details of clinic visits, that were paid for through this health wallet, were registered by clinic staff on the same digital platform (M-TIBA). Patients could buy their prescribed medications and other commodities such as BG strips from the clinic where they were enrolled. Stock management of these items was responsibility of the clinics and already part of their usual business before the care model was implemented.
- The opportunity to join monthly facility-based patient support groups

**Figure 1:**
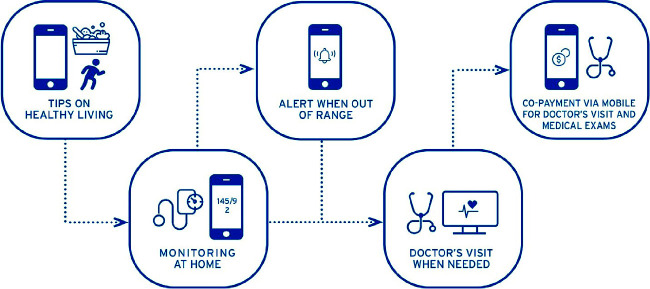
Integrated NCD management throughout a patient journey.

Physicians had access to a dashboard supporting them to identify at risk patients based on their self-measurement frequency and values entered. Financial data generated through the health-wallet and blood-pressure (BP) and BG data were used to analyze both care-utilization and costs under a co-payment model and outcomes of care to advocate such a mobile-based model to both public and private health insurers. Figure 1 provides an overview of the integrated NCD management throughout a patient journey.

**Table 1:** Profile of end-survey respondents.

	**Total**		**Hypertension (HTN)**		**Diabetes (DM)**		**Both HTN & DM**	
	**1045**		**566**	(54%)	**168**	(16%)	**311**	(30%)
**Age** (mean)	57.3		56.8		51.8		61	
**Sex**								
Males	339		179	(53%)	56	(17%)	104	(31%)
Females	706		387	(55%)	112	(16%)	207	(29%)
**Education**								
Tertiary	159		91	(57%)	26	(16%)	42	(26%)
Secondary	409		218	(53%)	71	(17%)	120	(29%)
Primary	430		235	(55%)	63	(15%)	132	(31%)
No formal education	47		22	(47%)	8	(17%)	17	(36%)
**Marital status**								
Married/living together	732		392	(54%)	125	(17%)	215	(29%)
Widowed	159		92	(58%)	17	(11%)	50	(31%)
Divorced/separated	88		47	(53%)	14	(16%)	27	(31%)
Never married	66		35	(53%)	12	(18%)	19	(29%)
**Occupation**								
Formal employment	74		46	(62%)	14	(19%)	14	(19%)
Informal employment*—*trader	366		193	(53%)	68	(19%)	105	(29%)
Other Informal employment	125		73	(58%)	24	(19%)	28	(22%)
Unemployed	480		254	(53%)	62	(13%)	164	(34%)
**County**								
Nairobi	613		295	(48%)	122	(20%)	196	(32%)
Kiambu	184		107	(58%)	28	(15%)	49	(27%)
Nyeri	128		90	(70%)	8	(6%)	30	(23%)
Vihiga	120		74	(62%)	10	(8%)	36	(30%)
**Residence**								
Rural	248		164	(66%)	18	(7%)	66	(27%)
Urban	797		402	(50%)	150	(19%)	245	(31%)

### Methods used to evaluate impact

Ethical approval for this evaluation study was obtained through Amref Health Africa ESRC. A mixed methods approach was used to evaluate implementation of the care-model with three data collection approaches. A survey was conducted with patients at baseline and project endline (i), self-measurement data was extracted from the Afya Pap database (ii) and health care utilization and savings data were extracted from the M-TIBA database (iii). Of the 1.626 patients that registered in the different clinics, a total of 1.278 patients were enrolled for follow-up and evaluation purposes. Between November and December 2020 (COVID-19 pandemic period), end-line survey data were collected of a total of 1.045 program participants through a phone-administered questionnaire. For 432 of the 1.045 study participants extensive baseline survey data were available as well—collected in September 2019.

### Quantitative survey data collection

A modified STEPS questionnaire was used to collect data at baseline during enrolment and at end line. The interview questions included demographics (age, sex, education, income, ethnicity), CVD risk factors (use of alcohol, smoking status, physical inactivity, unhealthy diet), anthropometric measurements (height, weight, waist circumference, arm circumference, hip circumference). The data for the end-line survey were collected by telephone due to COVID-19 pandemic interference. Since the patients followed to the end-line had BP and/or BG machines previously issued at the enrolment stage, they were requested to take their BP or BG measurements at the time of the phone interview to relay the information to the interviewer via SMS text. All responses to the questionnaire were electronically recorded on a tablet. To facilitate data collection over the phone, a shortened version of the STEPS survey was used. Body mass index (BMI) and waist-hip ratios were not measured during the exit survey as patients were not able to perform these measurements at home.

### Analysis

Descriptive statistics (proportions, means and standard deviations) were used to summarize patient’s background characteristics (socio- demographic information) as well as behavioral and anthropometric characteristics (e.g. smoking status, alcohol consumption, unhealthy diet, BMI, waist-hip ratio) and clinical measurements such as BP and BG. The number of days each patient measured their BP or BG as well as the number of times they participated in a patient- support group over the study period, was also calculated. Average monthly savings behavior and health care utilization were calculated through M-TIBA wallet transactions.

Changes in behavioral risk factors between baseline and follow-up time points was analyzed either using McNemar’s Chi-square test or paired sample t-test as applicable. Changes in BP and BG measurements over the follow up months were analyzed using paired sample t-tests. Odds ratios for reaching BP or BG control were calculated for joining support groups and levels of self-measurements, with correction for demographic and baseline characteristics using a Poisson regression model.

## RESULTS

### Patient characteristics

Table 1 shows the characteristics of all the 1045 respondents who were surveyed at the end of the project. Most patients had hypertension (84%), with 54% of patients having hypertension only and 30% having both hypertension and diabetes. Sixteen percent of patients had diabetes only. The patients had a mean age of 57.3 years; two-thirds (68%) were female. Nearly all (95%) had at least primary level education but 46% were unemployed; the majority (70%) were either married or living together and 76% resided in urban settings. Slightly more than half (59%) were recruited from Nairobi county, 18% Kiambu, 12% Nyeri and 12% Vihiga counties. Follow-up time per patient varied between 10 and 21 months (average 14 months). Out of the patients entering measurements, 45% was doing this through the smartphone application and 55% through the USSD-based menu for feature phones. Also notable is the bigger proportion of patients with hypertension in the rural population, which was as expected based on studies showing lower prevalence of diabetes in rural compared to urban settings in Kenya [9].

### Health outcomes

For 432 randomly selected patients, baseline data was available and could be compared to end-line survey data. In this population, proportion of patients with controlled BP- and BG-levels was significantly higher (*P* = 0.01) at endline survey compared to baseline: BP control-levels were 50% at end-line compared to 42% at baseline (McNemar’s chi square = 7, *P* = 0.01) and glycemic control was 74% at end-line compared to 57% at baseline (McNemar’s chi square = 8, *P* < 0.01). Patients who measured their BP at home and participated in peer support groups had a 21% higher likelihood of controlled BP compared to those who measure but did not participate in peer support groups (unadjusted OR 1.22 [95%-CI: 1.04–1.44]; see also: [26]). The prevalence of self-reported dietary risk factors such as harmful sugar (44% to 31%, McNemar’s chi square = 26, *P* < 0.01) and salt intake (37% to 26%, McNemar’s chi square = 8, *P* = 0.01) reduced significantly during follow-up among the patients who participated in peer support groups and among patients who performed home-based self-measurements.

### Acceptability and access to care

Generally, the model was acceptable with three-quarters (75%) of the patients engaging in self- monitoring using the devices provided to measure their BP or blood sugar at home and relaying their values via the Afya Pap smartphone application or USSD (SMS-based) data-entry tool; two-thirds (65%) saved funds on M-TIBA and about half (48%) participated in the peer support groups over the study period. Patients who never used the application provided the following reasons: lack of knowledge on the usage of the application (19% and 13% resp.), technical problems (5% and 6% resp.) and inadequate supply of consumable such as batteries, BP machines and strips for measuring glucose (3% and 6% resp.).

**Figure 2:**
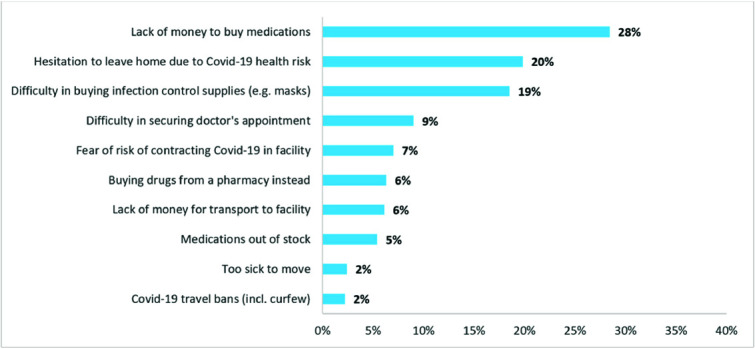
Reasons reported for unmet need to obtain primary care services in the last 6 months (% of respondents reporting this reason, based on qualitative categorization of responses).

Despite the highly reduced costs of care due to the co-payment model via M-TIBA, 48% of respondents reported an unmet need to obtain primary care services in the 6 months predating the end-line survey. The two most common reasons for unmet need of primary care are lack of finances to buy medications and perceived Covid 19 risks preventing people to seek care—either making people hesitant to leave their house or fear of contracting the virus in the facility (Fig. 2).

### Self-measurements

**Figure 3:**
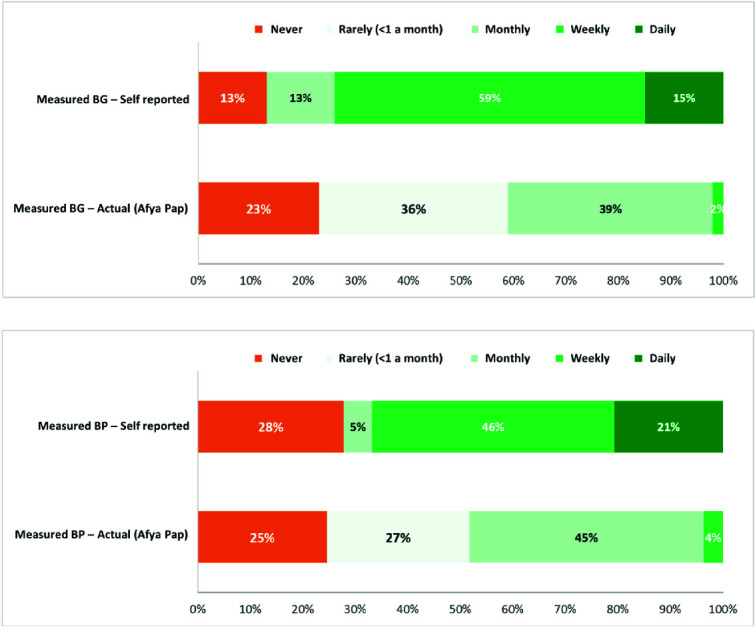
Frequency of BP measurements (top) and BG measurements (bottom) at home (self-reported versus actual entries in Afya pap, %).

40–50% of patients entered their measurements into the Afya Pap application at least on a monthly basis. A discrepancy was observed in self-reported frequency of measurements at home versus the frequency of measurement found in Afya Pap data (Fig. 3). Many respondents reported they were not always able to upload their measurements in the application due to either connectivity issues or timing out of the USSD-menu and only noted their measurements down in a booklet. Participation in the peer support groups increased the frequency of home-based self-monitoring of BP but not of BG measurements. Unfortunately, we were not able to stratify this analysis to rural and urban patients in our database. Based on qualitative observations of our project management staff, we do expect the use of the digital self-monitoring application to be lower in the clinics in Vihiga county.

### Healthcare savings

About two thirds (680 out of 1045) of surveyed participants saved on their M-TIBA wallet to pay for health care services. The median monthly savings (data was not normally distributed) on M-TIBA were 1.3 USD (25th percentile: 0.75 USD, 75th percentile: 5.2 USD) and was higher among patients who participated in peer groups compared to those who never participated (2 USD versus 0.45 USD).

### Healthcare utilization and costs

**Figure 4:**
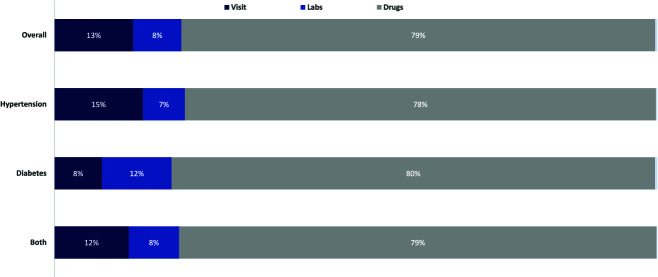
Proportion of spending on visit consultations, laboratory investigations and medicines (% by disease/risk factor type).

About two-thirds of patients (63%) attended at least one follow-up visit. Among those with at least one follow-up visit, average visit frequency was 1.3 visits per quarter. Average monthly total costs of care as registered on M-TIBA for these patients was 8.36 USD. With the lowest costs seen for those with hypertension only (6.54 USD per month), higher costs for those with diabetes only (9.70 USD per month) and highest costs for co-morbid patients (13.06 USD per month). Eighty percent of all costs was spent on medications (Fig. 4). Given the probably altered health care utilization patterns due to the COVID pandemic we expect this to be an underestimate. Additionally, not all costs of care were recorded via M-TIBA. Patients reported they would not always receive their care from the M-TIBA registered facility where they were enrolled but would e.g. at times get their medications from local pharmacies or attend public facilities for free consultations. Based on our data we were not able to do a thorough cost effectiveness analysis of the intervention as not all health care utilization costs were recorded and long-term data on reductions in complications was missing. Given the high undertreatment of many patients with NCDs, we expect implementation of new care-models that increase adherence to always be an additional investment instead of a short-term cost-saving strategy, although the long-term benefits of investing in chronic care in terms of health and economic improvements are evident [27].

## DISCUSSION

### Comparison to similar initiatives in the region

To our knowledge, no studies have evaluated integrated NCD care models that incorporate a digital approach to both care delivery and financing in the SSA region. A meta-analysis on mobile messaging-based interventions [28] and study on digitized linkage to care interventions [29] found no significant improvements, highlighting the need for more comprehensive digital models for NCD management. Several studies have demonstrated the effectiveness of partially digital, integrated care delivery models, primarily for hypertension, which have shown similar or better outcomes in achieving controlled values among patients [30–32]. The larger health impacts observed in some of these studies may be attributed to the greater involvement of research nurses who provided support and patient follow-up, a factor that was not included in our implementation to ensure sustainability and maintain a real-world setting without reliance on personnel that may not be available. Nonetheless, these findings underscore the crucial role of training and engaging healthcare workers, both within and outside the clinic, to ensure the success of digital integrated care models.

In terms of digital financing models, analyzing the quantifiable impact on healthcare access and outcomes is complex. Recent studies do consistently show that these technologies have the potential to improve the efficiency and transparency of health financing leading to more effective and equitable use of resources and contributing to UHC [33, 34]. Notable regional examples included in these studies are digital social health insurance in Kenya and the Community Based Health Insurance in Rwanda, both of which leverage digital platforms to enroll beneficiaries, verify eligibility and manage mobile payments. Achieving the benefits of digital health financing, however, depends on equitable access to digital infrastructure and technology, as well as sufficient levels of digital literacy—conditions that are often lacking. This highlights the critical need for strategic investment in both digital infrastructure and literacy programs, targeting healthcare workers and patients alike.

### Key insights and recommendations

Building upon the quantitative insights discussed above and drawing from qualitative perspectives gained through interactions with the implementation team, clinicians, patients, health insurers and policy makers, we have identified six pivotal learnings and accompanying recommendations.

First of all, **self-measurement is very feasible,** with more than 2/3rds of patients able and willing to measure their BP or BG levels independently or with help of an informal caregiver at home. Self-measurement helps to increase awareness of the nature of conditions like hypertension and diabetes and the relation between non-adherence and outcomes. It also empowers patients to take control of their disease. Combined with personalized feedback-messages and linkage to care to access clinic-led patient management and medications we expect this to have contributed to the higher control-rates seen in patients at follow-up, although our evaluation setup was not designed to show causality. Studies in several settings show that home- or community-based measurements embedded in a system of personalized feedback and clinic-led follow-up lead to better health outcomes [35–37]. More recent studies have also shown that implementation of digital self-management support through existing communication platforms (such as WhatsApp© or WeChat©) increases acceptability and effectiveness [38]. We did find that patients using the USSD-version of the self-measurement data-entry tool on feature phones (non–smartphones) frequently ran into challenges with timing out of the USSD-menu leading them to not submit measurements but only record on paper. In our experience non-smartphone individual data entry is not feasible. For those patients with only access to feature phones and those for whom it proves too complex to self-manage their condition at home, measurement within patient support groups or community-based follow-up through community health workers (CHWs) may be a better option. Also, patients in our program were provided with devices. Based on interviews we expect ~1/3rds of patients to be able to pay for their own device. For scalability of a self-monitoring approach, other models, such as measurement at pharmacies or in central places in the community should also be explored [39].

A second key learning is that **digital alone is not enough**. Besides digital support, face to face support is needed, especially for patients who struggle to use the digital tools. We partnered with the Kenya Defeat Diabetes Association and implemented their peer educator model in which one patient is trained to be an educator. During the patient group meetings, the peer educators take the group through a topic that they have agreed upon, such as how to use the digital tools or topics related to lifestyle. The healthcare provider also participates to engage with the patients and to get feedback from them on the services offered. Some groups also engage in income-generating activities to support their members and cover costs of operations. This model is very low costs and self-sustaining [40–43]. In certain settings it could be beneficial to incorporate outreach through CHWs to provide further home support to vulnerable patients that are not attending group meetings on a regular basis (see also Table 2).

**Table 2:** Summary of different approaches to integrated NCD-models along elements of the Wagner chronic care model and universal digital health functionalities required.

	Self-management support	Delivery system design	Decision support	Clinical information system	Healthcare organization	Community resources
*Initial integrated NCD-model as described in case study*	Digital lifestyle counseling, home based self-measurements and individualized feedback messages	Dedicated NCD-clinic days Remote monitoring of self-measurements Teleconsultations when possible	Risk stratification of patients based on measurements and care-adherence	Dashboard for proactive patient management with insight in self-measurements	Co-payment model for out-patient care services	Health education and lifestyle modification in community-based patient support groups
*Variation 1: low insurance coverage*	Same	Same	Same	Same	Group-based procurement of medications	Same + Financial support through contributions to community based patient support groups
*Variation 2: highly limited affordability & smartphone penetration*	Community-based measurements + SMS-based feedback	Same + training of CHWs to follow-up patients in the community	Same + risk stratification tool for CHWs indicating when referral is needed	Same	Group-based procurement of medications	Same + income generating activities in community groups
*Universal digital health functionalities according to WHO digital health classification*	1.1 Targeted client communication 1.4 Personal health tracking	2.4 Telemedicine	2.3 Healthcare provider decision support 2.6 Referral coordination	2.2 Client health records	3.5 Health financing (tracking and transmitting)	

Thirdly, **accessing medicines and teststrips (for those with diabetes) remains a key challenge** for patients and accounts for 80% of all costs of care to patients. Even in a co-payment model highly reducing the out-of-pocket financial barrier, almost 50% of patients reported issues with accessing care. This was partly due to challenges with available funds, opportunity-costs of traveling to their clinic and availability of needed medicines at a clinic level. Access to medicines and strips needs to be addressed in any integrated NCD care-model. This is especially the case in settings where initial BP or BG-values on diagnosis are relatively high and strict BP-control is essential to avoid complications, often making at least one oral medication necessary on top of lifestyle counseling. The wide generic availability of low-cost antihypertensive and oral antidiabetics does make this access possible at an acceptable price-level for many patients, but supply and procurement issues need to be addressed. An approach to this is joint procurement of medicines by patient support groups that also financially support each other to access these medicines. This strategy was implemented towards the end of this program to support patients to continue accessing care after financing for the co-payment model ended. Several patient groups are now experimenting with bulk purchasing of drugs at whole sales prices for the whole group. This is a model that is becoming increasingly prevalent and successful results have been reported in several studies [44, 45].

The **transition from demand-driven in-person care to proactive population-based patient management is complex for clinics**. In our implementation, we did not take enough account of challenges faced by clinics for this transition. The demanding nature of daily workload through busy waiting rooms leaves minimal room for the adoption of new operational paradigms. Connectivity issues and an insufficiency of digital hardware infrastructure within clinics pose substantial obstacles to the effective implementation of proactive follow-up protocols. Furthermore, the absence of financial reimbursement for the utilization of digital or mobile patient engagement tools introduces a notable financial constraint. Additionally, the lack of unanimous endorsement among clinic leadership teams regarding the merits of proactive care further complicates this shift. To address these challenges methodically, a structured approach to support clinics to pivot their way of working is needed, including a screening of their baseline situation. This requires both a significant allocation of resources and a commitment to investment at the leadership level to facilitate a seamless transition toward proactive patient management. On top of that we recommend healthcare payers to consider an incentivization model for partially digital care to make it economically appealing for healthcare facilities. When the clinical infrastructure is at a level of digital maturity where it is not feasible to provide pro-active patient management, another strategy is to outsource pro-active patient monitoring processes to a more centralized remote care provider.

Our fifth and profoundly significant learning underscores that **achieving financial sustainability for integrated NCD care models in Kenya is unlikely to materialize from the health insurance sector in the short term**. Our extensive engagement in workshops and interviews, involving both public health insurance entities and three private health insurers, delved into various approaches aimed at crafting bundled insurance products tailored for patients with chronic diseases, including the exploration of a co-payment model. With one insurer, we engaged in multiple follow-up discussions to assess the feasibility of developing such an insurance offering, yet a pilot stage was not reached. In our experience, the health insurance sector grapples with overarching issues pertaining to the financial robustness of their services. The adoption of digital technologies to enhance efficiency and expand coverage also remains a challenge. Given this landscape, we recommend a multifaceted approach to secure the financial sustainability of integrated NCD care models in Kenya. Firstly, exploring partnerships with public and private financing entities beyond traditional health insurers may provide alternative avenues for securing funding. Secondly, leveraging government-supported initiatives and international aid programs aimed at bolstering healthcare infrastructure and NCD management could be instrumental. In addition, using innovative financing tools to more efficiently utilize patient out-of-pocket spending should be explored to ensure optimal resource allocation and long-term viability of these models.

Lastly, there is **no one-size-fits-all implementation strategy** for integrated NCD-models. The best approach to self- or in community measurements, necessity of centralized remote care services and appropriate financing mechanism depends on the context of implementation. Rural and urban settings have their own specific challenges, but also within these settings high variation is present, for instance in terms of insurance coverage, digital infrastructure and smartphone penetration. The different implementation strategies can, however, be enabled with similar digital health infrastructures, especially when national governments invest in the groundwork for interoperability and data-sharing. Table 2 summarizes the initial design of the integrated NCD-model and two possible variations depending on the level of insurance access and digital capabilities of patients across elements of the Wagner chronic care model and core digital health classification functionalities needed to enable such an integrated NCD-model (WHO classification of digital health interventions v1.0). Research analyzing real-world datasets on efficacy of different implementation-models could help guide design of integrated care approaches for different target groups.

### Long-term impact & sustainability of integrated, digital NCD care models

Integrated digital models for managing NCDs in SSA hold significant promise for long-term impact and sustainability, particularly in the context of cardiometabolic multimorbidity. A key element of their effectiveness is the integration of chronic care approaches that bridge healthcare services across different levels of the health system, ensuring continuity of care and better outcomes for patients with complex NCDs like hypertension, diabetes and cardiovascular diseases. Research suggests that these models can lead to improved clinical outcomes, such as better BP and glycemic control [24]. The integration of mental health support also improves patients’ quality of life and medication adherence, further enhancing long-term disease management.

From a sustainability perspective, digital platforms that support remote monitoring, data collection and telemedicine are key components. These platforms help overcome geographical and resource constraints common in SSA, making healthcare more accessible and efficient. Furthermore, by streamlining communication between healthcare providers and patients, digital models reduce the burden on overstretched health systems, allowing for scalable interventions that can be sustained even in resource-limited settings. Recent reports by WHO underline the significant long-term impact that can be made by investing in digitally supported NCD care models, estimating that an additional 0.24 USD per patient per year will save over 2 million lives and have almost 200 billion USD in economic benefits in the next ten years [46]; (WHO, 2021) [47]. However, challenges such as infrastructure limitations, unintegrated systems, digital literacy and the need for continuous healthcare worker training must be addressed to ensure the long-term success of these models [48].

## CONCLUSION

In conclusion, the escalating burden of NCDs in LMICs requires urgent attention. These chronic conditions account for a significant proportion of global deaths, with high numbers of premature fatalities occurring within LMICs. Chronic care occupies a central role in achieving UHC by recognizing the unique challenges posed by long-term health conditions and implementing patient-centered approaches that empower individuals to manage their health effectively. Embracing chronic care as an integral component of UHC advances the collective vision of a healthier, more equitable world where no one is left behind in their pursuit of well-being. Adherence to medication and lifestyle modification are crucial to manage NCDs and prevent complications. Hence, the goal should be to support patients to take control of their own health, embedded in an informal and professional care network that can intervene and support when needed. Digital approaches are uniquely suitable to facilitate this. Furthermore, in a context where health financing is severely constrained and will likely remain so in the foreseeable future, efficiencies in healthcare delivery and procurement matter when it comes to giving more patients access to the care they need. The case study presented highlights the potential of digital and mobile-based care models to improve access and quality of care for hypertensive and diabetic patients in Kenya, underscoring the importance of multifaceted solutions in the pursuit of Universal Health Coverage and effective NCD management.

## Supplementary Material

250106_Rethinking_chronic_care_Supplementary_materials_oqaf002

## Data Availability

The data underlying this article were provided by CarePay© and Baobab Circle© under a data-sharing agreement with PharmAccess for research and project-management purposes. Data will be shared on request to the corresponding author with permission of all parties.
