# Supplementary material for: Rethinking chronic care: how blended patient-centered care delivery and innovative financing models can contribute to achieving universal health coverage—a case study of an integrated approach in Kenya
Source: Oxf Open Digit Health. 2025 Jan 12;3:oqaf002. doi: 10.1093/oodh/oqaf002 (PMC11932143; doi:10.1093/oodh/oqaf002)
Supplement: 250106_Rethinking_chronic_care_Supplementary_materials_oqaf002 [file 250106_Rethinking_chronic_care_Supplementary_materials_oqaf002.docx]

### **Supplementary materials**

### **Rethinking chronic care: How blended patient-centered care delivery and innovative financing models can contribute to achieving Universal Health Coverage – a case study of an integrated approach in Kenya**

Judith van Andel^1,2,*^, Gloria P. Gómez-Pérez^1,2^, Peter Otieno^3^, Angela Siteyi^1^, Julia Teerling^1^, Tobias Rinke de Wit^1,2^, Gershim Asiki^3^

^1^ PharmAccess, Amsterdam, The Netherlands & Nairobi, Kenya

^2^ Amsterdam Institute of Global Health and Development, Amsterdam, The Netherlands

^3^ African Population Health Research Center, Nairobi, Kenya

*Corresponding author: [judithvanandel@gmail.com](mailto:judithvanandel@gmail.com); Paasheuvelweg 25, PO Box 22700, 1100 DE Amsterdam, The Netherlands

**
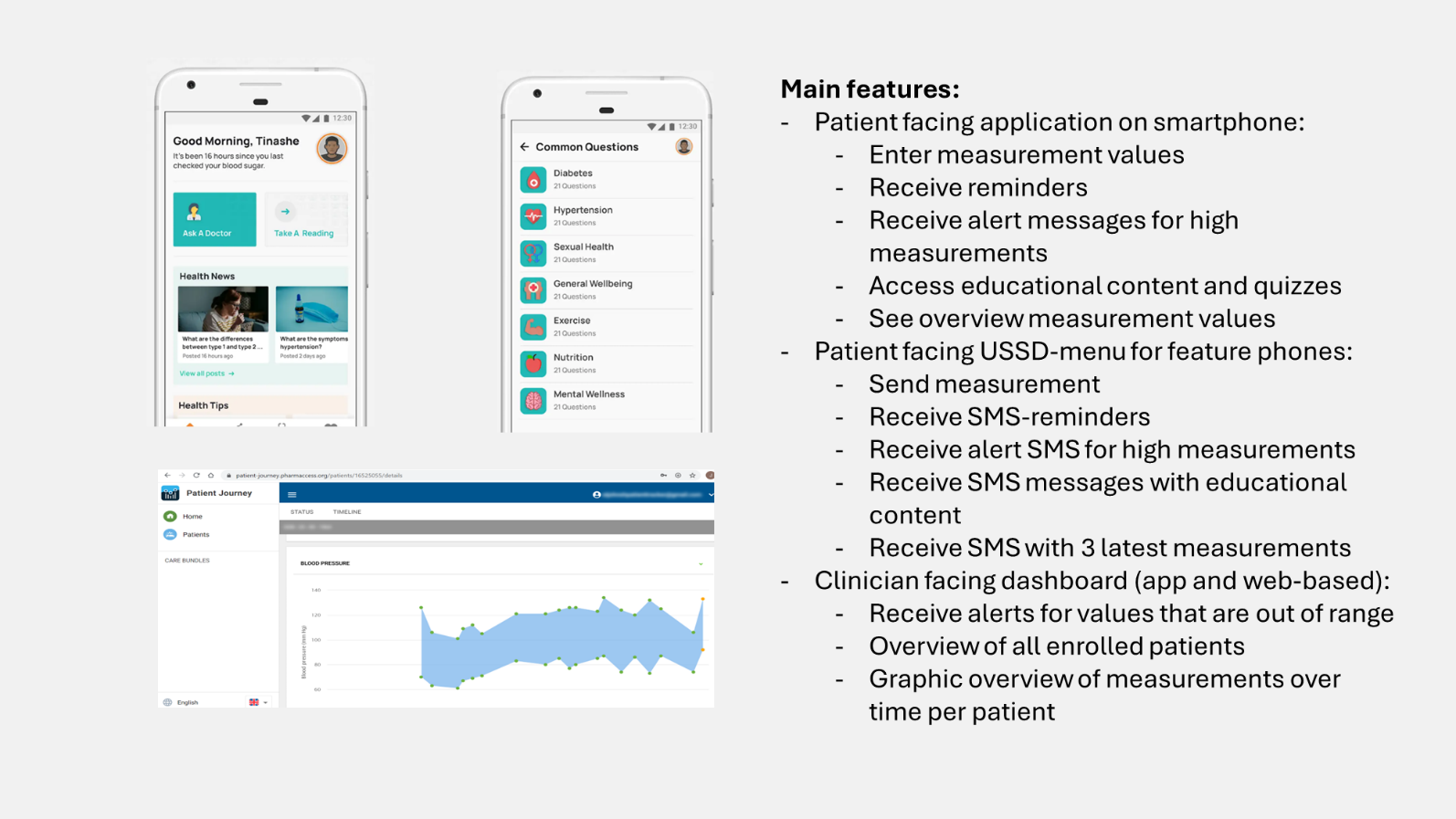
**

Supplementary figure 1: Main features of the Afya Pap application (Baobab Circle©) to monitor blood pressure and blood sugar levels
